# Dual Infection by Dengue Virus and *Shigella* s*onnei* in Patient Returning from India

**DOI:** 10.3201/eid0902.020026

**Published:** 2003-02

**Authors:** R. N. Charrel, M. Abboud, J. P. Durand, P. Brouqui, X. De Lamballerie

**Affiliations:** * Laboratoire de Bactériologie-Virologie, Marseille, France; † Faculté de Médecine, Marseille, France; ‡ Chemin des Bourrelys, Marseille, France

**To the Editor:** Two days after returning from a 4-week trip in India, a 44-year-old woman was admitted to a local French hospital with diarrhea and a fever (40°C). The fever had started 2 days before her return and was associated with myalgia and backache. The patient had not been vaccinated against yellow fever and did not take malaria prophylaxis during her trip. Blood smears were negative for malaria parasites. Biological analyses (complete blood count, liver enzymes, urine culture, stool culture, blood cultures) were ordered. She was sent home with an empirical treatment with ofloxacin (200 mg per day). Biological parameters were within the normal range, except for her platelet count, which was at the lower limit (170 G/L). Microbiologic analyses of stools yielded an isolate of *Shigella sonnei* serotype 9.

One week later, the patient was admitted to the infectious diseases unit of a university hospital in Marseilles, France, with persistent fever, myalgia, and a 7-kg weight loss; she had no digestive manifestations. Results of viral serology tests were negative, except that immunoglobulin (Ig)M, but not IgG, specific for dengue virus (formal name: *Dengue virus*; [DENV]) was present. This result was obtained with the Dengue Virus IgM and IgG Rapid Immunochromatographic Card Test (Biotrin, PanBio Pty. Ltd., Brisbane, Australia) and was confirmed by the Dengue Duo IgM-capture and IgG-capture enzyme-linked immunosorbent assay (ELISA) (PanBio) and a previously described in-house IgM antibody capture ELISA tests (*1*). Forty days later, a second serum specimen was collected and tested positive for DENV IgG with the persistence of IgM by three techniques. In light of these results, the diagnosis of primary dengue infection was established, according to criteria of the Centers for Disease Control and Prevention (*2*). A literature review did not find any documented case of coinfection by DENV and *Shigella*.

In India, DENV causes epidemic and sporadic cases year-round, with a peak in frequency from August to November, during the humid season (*3*). During the patient’s trip, she successively visited Mumbai (Bombay); went north to the Shimla district, Himachal Pradesh, the Agra district, and Uttar Pradesh; and came back to Bombay 3 days before leaving for France. If one assumes a 3- to 6-day incubation period, she likely acquired the dengue infection in Uttar Pradesh; the virus has been reported to circulate in this area (*4*).

Although severe forms are increasingly reported, most cases of dengue fever consist of a mild illness with nonspecific symptoms such as headache, myalgia, and malaise. Dengue fever may go underdiagnosed in travelers returning from DENV-endemic areas. This case underlines the importance of a thorough interview and clinical examination to detect characteristic signs (photophobia, painful ocular mobilization, skin rash) in patients returning from areas endemic for dengue fever, when clinical and biological signs incompletely correlate with the primary diagnosis. Since dengue fever is the second most frequent cause of febrile illness in persons returning from tropical areas, such patients should be routinely screened for the disease.
